# The effects of dynamic, static, and proprioceptive neuromuscular facilitation stretching on local & non-local range of motion and pain onset

**DOI:** 10.1371/journal.pone.0358885

**Published:** 2026-09-21

**Authors:** Hunter Parsons, David G. Behm

**Affiliations:** School of Human Kinetics and Recreation, Memorial University of Newfoundland, St. John’s, Newfoundland and Labrador, Canada; Adnan Menderes Universitesi, TÜRKIYE

## Abstract

This study compared the immediate effects of a single 30-second stretching bout on local (stretched) and non-local (non-stretched) range of motion (ROM), pain sensitivity/onset, strength, and endurance. Fourteen participants were involved with this within-subjects, repeated-measures study, consisting of four conditions: 30-seconds of static (SS), proprioceptive neuromuscular facilitation (PNF), dynamic (DS) stretching, and a no-stretch control condition. Passive hip flexion and shoulder extension ROM, pain pressure thresholds (PPT), knee flexors and right shoulder extension maximum voluntary isometric contraction force (MVIC), 30-s MVIC fatigue index and muscle activation (electromyography: EMG) were measured pre- and post-intervention. The primary findings were that 30-seconds of SS elicited greater moderate magnitude ROM improvements in the stretched limb (p < 0.001, 8.1%) compared to control. Non- significant (p = 0.08) SS-induced ROM gains were observed with the contralateral leg inferring the possibility of crossover effects in some individuals. There were no significant effects on the shoulder with any intervention. All conditions including control experienced MVIC force decreases with the stretched limb (p = 0.007). However, there were no significant PPT interactions. Women demonstrated greater ROM whereas men exhibited greater force, EMG activity, and fatigue indices. In conclusion, while passive ROM improved across all conditions, these gains can be accompanied by declines in MVIC force. Overall, 30-seconds elicited significant increases in ROM of the stretched leg respectively; however, it failed to produce any significant effects on the contralateral leg and shoulder.

## Introduction

The literature has shown that an acute session of static stretching (SS), proprioceptive neuromuscular facilitation (PNF) stretching, and dynamic stretching (DS) can increase or improve range of motion (ROM) [1–3]. While often used as a tool in warm-up routines and rehabilitation, there remains controversy in which method may result in the greatest acute ROM improvements [1,2]. Studies comparing these modalities have reported conflicting results, with some suggesting PNF elicits the most significant gains partially due to greater reflex activity such as autogenic and reciprocal inhibition [2,4,5], while others indicate that SS and PNF produce no significant differences in ROM [6,7].

Stretching has also been shown to produce measurable changes to pain perception through increases in pain pressure threshold (PPT) [8–11]. This increase in pain (stretch) onset and tolerance has been identified as a mechanism underlying increases in ROM [2,12,13]. Furthermore, this suggests that acute ROM improvements may be driven more by changes in sensation or increased stretch tolerance than by purely mechanical changes in the muscle-tendon unit, implying activation of pain-modulating pathways [2,8–13]. Studies interested in this mechanism have reported non-local adaptations, in which stretching a single limb has been observed to increase ROM and/or PPT in non-stretched muscle groups [14,15]. These systemic effects are potentially mediated by mechanisms such as diffuse noxious inhibitory control (DNIC) effects [16,17] and the gate control theory [8,18].

Despite the current body of literature on stretching, gaps remain regarding the influence of different modalities on these acute non-local adaptations in ROM [19] and PPT [20]. Hadjizazdeh Anvar et al. [21] indicated that the afferent excitability of the spinal motoneurons and corticospinal excitability may not play a substantial role in non-local muscle’s ROM or force output responses. However, while some studies observe global hypoalgesia following stretching [15,22], the specific dose of stretching, as it relates to intensity and duration, needed to activate supraspinal inhibitory pathways is not well defined. Further, while research on performance decrements has largely focused on prolonged stretching durations (>60-seconds per muscle group), shorter acute durations (<60-seconds) remain underexamined [1–3,8]. Although prolonged SS durations are linked to reduced force output and fatigue, clarity is lacking regarding the impact of shorter bouts, as well as the performance costs of DS and PNF [1,23,24]. This leaves 30-second intervals of SS, DS, and PNF under-examined, especially when comparing them in terms of acute effects on maximum voluntary isometric contraction (MVIC) force, electromyography (EMG), and 30-s MVIC endurance.

Thus, the purpose of this study was to compare the immediate effects of a single 30-second bout of SS, DS, and PNF on local and non-local ROM and PPT. Additional areas of interest included comparing the effects of these modalities on MVIC force, EMG activity, and 30-s MVIC endurance (fatigue index). It was hypothesized that all modalities would increase ROM and PPT, with SS yielding the greatest ROM gains.

## Methods

### Experimental design

Fourteen (14) participants were recruited for this study between January 6, 2026, and April 24, 2026. The within-subjects, repeated-measures experimental design consisted of four conditions: SS, PNF, DS, and a no-stretch control condition. Each condition was assigned a number from 1 to 4, and then an online random number generator was used to select the session intervention. Each session began with a standardized 5-minute warm-up on a cycle ergometer (Monark® cycle ergometer, Monark, Stockholm, Sweden) at 60–70 RPM and 1 kilopond resistance. During each participant’s first visit to the laboratory, the first 20-minutes were used as a familiarization period for procedures and to answer any questions. Height and weight were also measured during this time. Passive ROM of both hips (hip flexion) and right shoulder (shoulder extension), PPT of both hamstrings and right deltoid, both knee flexors MVIC and right shoulder extension MVIC, and fatigue index and muscle activation (EMG) were tested. ROM, PPT, MVIC force and EMG were measured pre- and post-intervention. The 30-s MVIC tests of the stretched leg, contralateral leg, and right shoulder (laboratory equipment restrictions only allowed testing of the right ipsilateral shoulder) were performed in a randomized order, using the same randomization method as the intervention. The 30-s MVIC tests were performed last and post-intervention only, as testing endurance pre-test would significantly affect all other measures.

### Participants

Seven (7) healthy men (mean ± SD; 21.57 ± 1.13years, 180.91 ± 8.17 cm, 88.64 ± 13.15 kg) and 7 healthy women (mean ± SD; 21.86 ± 1.21years, 165.74 ± 7.41 cm, 70.64 ± 5.39 kg) participated in this study and were all recreationally active at least two times a week. All participants were right hand and leg dominant as evidenced by the hand used to write their signature and the leg used to kick a ball [19–21]. Participants completed the Physical Activity Readiness Questionnaire for Everyone (PAR-Q+) [25,26] to assess physical readiness. Individuals with current or recent musculoskeletal, neurological, cardiovascular, or rheumatological conditions were excluded, as well as those who use pain medication or have a history of chronic pain or diagnosed mobility impairments. Participants were instructed not to engage in intense physical activity 24-hours prior to their participation session to mitigate confounding variables. Participants were not informed of the study’s hypothesis. The study was approved by the institutional ethics board: Interdisciplinary Committee on Ethics in Human Research (20251197-HK) and in accord with the Declaration of Helsinki. All participants were verbally informed of the procedures and risks as well as reading and signing a fully disclosed consent form before commencing the experiment.

### Independent variables

For the SS protocol, the participants laid supine, relaxed, on a padded massage table. The investigator then moved the hip joint into flexion (to stretch the hamstrings) to the point of maximal discomfort (end range). This position was held for 15-seconds, the participants were given 15-seconds rest, then the investigator maintained the stretched position for an additional 15-seconds. The PNF protocol followed the same setup as the SS protocol. The researcher passively flexed the hip to the participant’s point of maximal discomfort and held for 5-seconds, followed by a near-maximal hamstrings contraction against the researcher for 5-seconds. The researcher then increased hip flexion to a new point of maximal discomfort and held for another 5-seconds. After 15-seconds of rest, the protocol was repeated once more. For the DS protocol, the participants were instructed to dynamically flex and extend their right leg as high as possible for 15 kicks (under control), following the tempo of a 60 beats per minute (bpm) metronome, reaching the apex of each kick at the end of each second. This was followed by 15-seconds of rest, then an additional 15 kicks. For the control intervention, participants’ measurements were gathered, and they were then told to sit and relax in a chair for 2-minutes.

### Dependent variables

Pre-intervention measurements followed the order of (1) ROM, (2) PPT, and (3) MVIC. Post-intervention followed the same order with the addition of a 30-s MVIC protocol (final measure). Two trials were performed for each limb of interest prior to intervention. If the second trial was 5% greater than the first, a third was performed, and the highest value was recorded. There were 15-seconds of rest given between the ROM and PPT trials, with at least 2-minutes of rest between MVIC trials. Post-intervention, only one trial was recorded per limb and measurement.

Passive hip joint (hip flexion, hamstrings) and right shoulder joint (extension) ROM were measured using a handheld electric goniometer (EasyAngle®, Meloq, Stockholm, Sweden). Testing of both hamstrings involved the goniometer placed on the lateral side of the knee, aligned straight with the malleolus, and held in place by the investigator. Participants were instructed to lie supine on a padded plinth, and the investigator moved their leg into flexion while ensuring the opposite leg remained on the surface by placing their knee on the participant’s thigh. Participants were instructed to keep the leg straight (knee extension) during measurement, signaling to the investigator when they reached the maximal point of discomfort (end ROM). Careful attention was paid to the goniometer during this time to ensure it did not move out of position.

Due to laboratory equipment restrictions, only the right shoulder was tested. For the right deltoid, the goniometer was placed on the anterior aspect of the participant’s wrist, aligned with the shoulder, and held in place by the investigator. Participants were stabilized by standing erect and bracing their backs against a support beam, allowing their right arm to have full sagittal plane ROM. They were instructed to keep their chest raised and use their opposite hand to anchor themselves firmly on the other side of the support beam. With their right arm in line with their hip, the investigator then pulled posteriorly (into extension), and the participant signaled when they reached the point of maximal discomfort. Careful attention was paid to the goniometer during this time to ensure it did not move out of position.

PPT was measured using a handheld pressure algometer (Pain Test^TM^ FPX, Wagner Instruments, Greenwich, CT, USA). For the hamstrings, participants were instructed to lie prone on a padded plinth. The investigator palpated for the semitendinosus muscle, beginning near the popliteal space and moving up towards the muscle belly where the tendon and muscle fuse (musculotendinous junction), and marked that point. Thereafter, the investigator pushed the tip of the pressure algometer into the marked point and instructed the participant to signal when they reached the onset of pain. For the right deltoid, participants were instructed to stand erect and brace their left side against a support beam. Participants were then asked to horizontally abduct the right arm against the investigator’s resistance so the investigator could mark the point at which the lateral deltoid connected to the humerus (musculotendinous junction). From there, the investigator applied pressure with the pressure algometer to the marked point and instructed the participant to signal when they felt the onset of pain.

MVIC force measurements were performed for both hamstrings and right shoulder. For the hamstrings, participants were seated and strapped to ensure stability, with arms crossed over the chest and the knee flexed at 120°. The ankle was secured with a padded ankle cuff attached to a strain gauge (Omega Engineering Inc., LCCA 250, 500 pounds; sensitivity = 3 mV/V, OEI, Don Mills, Ontario), fixed to an immovable anchor in the wall. Participants then maximally flexed the knee (contracted the hamstrings) for 4-seconds. Force signals were amplified 1000X and sampled at 2000 Hz, digitally converted (Biopac Systems Inc. DA 100 and analog-to-digital converter MP100WSW; Holliston, MA). For the shoulder, participants stood erect with their right hand in a padded cuff attached to the strain gauge, while using their left hand as a brace on the wall directly in front of them. Participants then pulled with maximal effort into shoulder extension, pulling their right hand in line with their hip. Participants were instructed to avoid leaning back during shoulder extension to avoid inadvertent force increases.

Muscular activity was monitored via EMG during MVIC and 30s MVIC measurements. Surface electrodes (Kendall® Medi-trace 133 series, Ag/AgCl, Chicopee, MA) with an edge-to-edge inter-electrode spacing of 20 mm were placed on the middle of the muscle bellies of the lateral deltoid (midway between the acromioclavicular joint and the deltoid insertion on the humerus) and biceps femoris (midway between the popliteal space and the gluteal fold). Ground electrodes were placed on the fibular head (for the lower body) and the acromion (for the upper body). Before electrode placement, the skin was shaved, prepared using an abrasive skin prep gel (Nuprep, Weaver and Company, Aurora, CO, USA), and cleaned with an isopropyl alcohol swab. EMG activity was digitally filtered with a linear phase Blackman −61 dB band-pass filter between 10 and 500 Hz, amplified (× 1000) and analog to digitally converted (12 bit). All EMG signals were recorded (Biopac System Inc., DA 100: analog-digital converter MP150WSW; Holliston, Massachusetts) with sampling rate of 2000 Hz using a commercially designed software program (AcqKnowledge III, Biopac System Inc.).

The 30-s MVIC measure was tested post-test only, after all other measurements were taken. The setup was identical to the MVIC measure for both the hamstrings and the shoulder. Participants were instructed to perform a 30-seconds MVIC, after which they were given 3-minutes of rest between the subsequent 30-seconds MVIC protocols for the other randomized limb tests.

### Statistical analysis

Statistical analyses were performed using IBM SPSS Statistics (Version 29.0; IBM Corp., Armonk, NY, USA). Microsoft Excel (Microsoft 365; Microsoft Corp., Redmond, WA, USA) was utilized for data visualization. The normality of the data distribution for all variables was assessed using the Shapiro–Wilk test. To analyze ROM, PPT, MVC forces and EMG, a two-way (4 × 2) repeated-measures analysis of variance (ANOVA) was conducted to determine differences between conditions (SS, PNF, Dynamic, Control) and time (pre- and post-test), as within-subject factors with sex as a between subject factor. Furthermore, a one-way ANOVA with sex as a between subject factor was performed for the 30-s MVIC fatigue index (Fatigue index = ((last 5 seconds of the 30s endurance set / first 5-seconds of the 30-s endurance set) – 1) x 100) for MVC forces and EMG. When significant main effects or interactions were observed, the Bonferroni post hoc test was employed to identify specific pairwise differences. Main effect and interaction effect sizes were assessed using partial eta squared (ηp^2^). Partial eta^2^ values represent effect size magnitudes as follows: 0.01: small, 0.06: medium, 0.14 or higher: large. Cohen’s d [27] effect sizes were calculated for individual differences or comparisons with <0.2: trivial, 0.2 – < 0.5: small, 05 – < 0.8: moderate and ≥0.8 representing large magnitude respectively. Statistical significance was accepted at p ≤ 0.05 for all analyses. The reliability of pre-test measures was calculated with intraclass correlation coefficients (ICC: Cronbach alpha model, type: consistency, two-way mixed, 95% confidence interval). Minimal detectable differences (MDD) were calculated with the following equation: MDD = SEM × z × √2

## Results

### Reliability (intraclass correlation coefficients)

ICC comparing the reliability of pre-test measures of the SS, PNF, Dynamic, and Control conditions for the stretched leg, non-stretched leg, and shoulder were as follows: ROM: 0.97, 0.98, and 0.96, PPT: 0.90, 0.86, and 0.96, MVIC force: 0.96, 0.96, and 0.91, MVIC EMG: 0.75, 0.73, 0.84 respectively.

### Range of motion

A condition x time interaction (F_(3,36)_ = 6.84, p < 0.001, 𝜂𝑝^2^ = 0.36) demonstrated that the SS post-test exceeded the control post-test by a moderate magnitude effect size (d = 0.55) of 8.1% with the stretched leg (Table 1). There were no further significant interactions. A non-significant main effect for condition (F_(3,36)_ = 2.54, p = 0.071, 𝜂𝑝^2^ = 0.175) was observed with the stretched leg with SS exceeding Control (p = 0.04) by 4.2% (d = 0.31). A main effect for time (F_(1,12)_ = 4.78, p = 0.049, 𝜂𝑝^2^ = 0.285) revealed an overall 2.2% (d = 0.23) increase in stretched leg ROM from pre- to post-test.

**Table 1:** Means ± Standard deviations for stretched leg. Asterisks (*) illustrate significant condition x time interaction differences between the two boxes in the same row. Hashtags (#) show significant MVIC pre- to post-test differences. Numbers in brackets indicate the minimum pre- to post-test detectable difference.

Combined (14)	SS pre-	SS post-	PNF pre-	PNF post-	DS pre-	DS post-	Control pre-	Control post-
ROM (^o^) Condition x Time p < 0.001	106.7 ±10.7	113.1 ±12.7 * (12.03)	105.5 ±10.6	109.5 ±11.9 (10.98)	108.4 ±10.1	108.8 ±10.3 10.1	105.8 ±11.5	104.5 ±11.1 * (10.8)
PPT (N)	115.7 ±62.0	126.6 ±61.6 (33.03)	111.3 ±53.8	121.5 ±61.1 (32.1)	119.9 ±67.2	130.1 ±65.1 (37.5)	123.4 ±66.5	120.5 ±63.4 (31.5)
MVIC (N) p = 0.007	306.05 ±51.2	288.1 ±46.3 # p = 0.045 (46.4)	314.6 ±66.1	273.7 ±53.6 # p = 0.003 (50.4)	301.8 ±64.8	287.5 ±50.3 # p = 0.05 (53.3)	306.8 ±65.3	290.0 ±58.6 # p = 0.038 (55.7)
EMG (V)	0.16 ±0.06	0.14 ±0.05 (0.048)	0.14 ±0.05	0.12 ±0.04 (0.037)	0.14 ±0.04	0.13 ±0.04 (0.05)	0.135 ±0.06	0.135 ±0.065 (0.05)
FI (%)		−28.9 ±9.3		−23.8 ±12.9		−26.4 ±12.1		−20.9 ±13.5
**Males** (7)	SS pre-	SS post-	PNF pre-	PNF post-	DS pre-	DS post-	Control pre-	Control post-
ROM (^o^)	97.7 ±13.2	103.0 ±15.2 *	97.4 ±10.6	101.4 ±16.3	100.1 ±11.4	100.1 ±12.1	97.4 ±13.9	95.2 ±13.8 *
PPT (N)	139.3 ±95.6	147.6 ±86.4	122.9 ±82.1	137.1 ±94.9	138.2 ±101.0	155.4 ±96.5	137.5 ±98.4	131.2 ±92.5
MVIC (N)	352.2 ±49.8	330.9 ±46.5 #	360.5 ±57.3	316.7 ±57.0 #	355.2 ±59.5	338.0 ±38.9 #	356.9 ±63.8	338.2 ±63.7 #
EMG (V)	0.18 ±0.07	0.16 ±0.06	0.18 ±0.07	0.15 ±0.05	0.18 ±0.04	0.17 ±0.07	0.14 ±0.05	0.15 ±0.07
FI (%)		−34.1 ±7.7		−31.6 ±9.3		−36.6 ±14.3		−23.1 ±15.6
**Females** (7)								
ROM (^o^)	115.7 ±8.2	123.1 ±10.2 *	113.5 ±10.6	117.7 ±7.6	116.7 ±8.7	117.5±8.5	114.3 ±9.1	113.8 ±8.3 *
PPT (N)	92.1 ±28.4	105.7 ±36.9	99.8 ±25.6	105.8 ±27.4	101.6 ±33.4	104.8 ±33.8	109.4 ±34.6	109.8 ±34.3
MVIC (N)	259.9 ±52.6	245.3 ±46.0 #	268.7 ±75.0	230.8 ±50.2 #	248.4 ±70.1	237.1 ±61.8 #	256.8 ±66.8	241.8 ±53.6 #
EMG (V)	0.14 ±0.05	0.12 ±0.04	0.10 ±0.02	0.10 ±0.03	0.09 ±0.04	0.09 ±0.02	0.13 ±0.07	0.12 ±0.06
FI (%)		−23.7 ±10.8		−16.1 ±16.6		−16.3 ±9.8		−18.7 ±11.3

Acronyms: SS: static stretching, PNF: proprioceptive neuromuscular facilitation, DS: dynamic stretching, ROM: range of motion, PPT: pain pressure threshold, MVIC, maximum voluntary isometric contraction, N: Newtons, EMG: electromyography, V: volts, FI: fatigue index.

While there was no significant interaction, the non-stretched contralateral leg also exhibited a non-significant main effect for condition (F_(3,36)_ = 2.44, p = 0.08, 𝜂𝑝^2^ = 0.169) with SS ROM exceeding PNF, DS, and Control conditions by 3.3% (d = 0.26), 1.5% (d = 0.13), and 3.1% (d = 0.26) respectively (Table 2). There were no significant findings for shoulder ROM.

**Table 2:** Means ± Standard deviations for non-stretched leg. A plus or addition sign (+) illustrates a main effect for condition with SS exhibiting significantly (p = 0.024) greater fatigue index deficits effects than PNF (sex data combined). An asperand (&) indicates a condition x time x sex interaction for the non-stretched leg showing that women had significantly greater ROM than men with every condition and time apart from non-significant differences with SS post-test (p = 0.07), and Dynamic post-test (p = 0.12). Numbers in brackets indicate the minimum pre- to post-test detectable difference.

Combined (14)	SS pre-	SS post-	PNF pre-	PNF post-	DS pre-	DS post-	Control pre-	Control post-
ROM (^o^)	105.5 ±10.1	106.7 ±8.9 (9.76)	102.7 ±9.2	102.5 ±12.4 (10.98)	104.6 ±9.6	104.4 ±10.0 (10.01)	104.0 ±9.8	101.9 ±10.0 (10.78)
PPT (N)	110.5 ±29.0	110.5 ±29.4 (28.8)	102.1 ±35.9	110.0 ±35.5 (28.7)	117.1 ±43.8	122.5 ±38.3 (32.3)	117.1 ±38.7	111.2 ±40.1 (29.2)
MVIC (N)	303.0 ±50.2	286.9 ±43.2 (45.8)	307.9 ±44.1	282.3 ±59.1 (50.3)	293.6 ±52.9	286.2 ±46.8 (54.1)	294.7 ±51.5	283.8 ±47.8 (52.7)
EMG	0.14 ±0.04	0.125 ±0.04 (0.03)	0.125 ±0.05	0.125 ±0.04 (0.03)	0.115 ±0.04	0.125 ±0.045 (0.04)	0.115 ±0.045	0.11 ±0.045 (0.03)
FI (%)		−31.3 + ±9.2		−19.6 + ±7.5		−27.8 ±12.6		−23.6 ±9.6
**Males** (7)	SS pre-	SS post-	PNF pre-	PNF post-	DS pre-	DS post-	Control pre-	Control post-
ROM (^o^) &	97.0 ±12.0	100.3 ±14.5	94.8 ±8.5	93.7 ±15.3	95.8 ±9.8	99.8 ±13.1	96.0 ±10.3	95.0 ±10.9
PPT (N)	133.7 ±42.3	138.5 ±36.5	122.0 ±55.7	120.5 ±46.3	135.9 ±50.1	144.7 ±43.6	128.7 ±43.8	118.1 ±41.2
MVIC (N)	349.3 ±44.2	331.1 ±38.1	344.3 ±42.0	318.0 ±66.4	348.6 ±42.3	341.7 ±43.0	330.1 ±39.3	336.5 ±47.1
EMG (V)	0.15 ±0.05	0.14 ±0.05	0.14 ±0.06	0.14 ±0.05	0.13 ±0.06	0.14 ±0.06	0.11 ±0.04	0.11 ±0.04
FI (%)		−34.6 ±11.4		−26.4 ±11.1		−33.1 ±11.2		−23.7 ±8.7
**Females** (7)								
ROM (^o^) &	114.0 ±8.1	113.2 ±9.3	110.7 ±10.0	111.4 ±9.5	113.4 ±9.4	109.1 ±6.9	112.0 ±9.4	108.8 ±9.1
PPT (N)	87.2 ±15.7	99.0 ±22.2	82.1 ±16.2	99.5 ±24.7	98.2 ±37.6	100.3 ±33.1	105.4 ±33.7	104.3 ±38.9
MVIC (N)	256.7 ±56.2	242.7 ±48.4	271.6 ±46.1	246.6 ±51.7	238.7 ±63.5	230.7 ±50.7	259.3 ±63.7	231.2 ±48.5
EMG	0.13 ±0.03	0.11 ±0.03	0.11 ±0.04	0.11 ±0.03	0.10 ±0.02	0.10 ±0.03	0.12 ±0.05	0.11 ±0.05
FI (%)		−28.0 ±6.9		−12.8 ±4.0		−22.5 ±14.0		−23.6 ±10.5

Acronyms: SS: static stretching, PNF: proprioceptive neuromuscular facilitation, DS: dynamic stretching, ROM: range of motion, PPT: pain pressure threshold, MVIC, maximum voluntary isometric contraction, N: Newtons, EMG: electromyography, V: volts, FI: fatigue index

Women exceeded men’s ROM for the stretched leg (F_(1,12)_ = 10.1, p < 0.001, 𝜂𝑝^2^ = 0.99, 17.5%, d = 1.75) and non-stretched leg (F_(1,12)_ = 8.40, p < 0.001, 𝜂𝑝^2^ = 0.993, 15.5%, d = 1.51). A condition x time x sex interaction for the non-stretched leg illustrated that women had significantly greater ROM with every condition and time apart from non-significant differences with SS post-test (p = 0.07), and Dynamic post-test (p = 0.12).

### Pain pressure threshold (PPT)

There were no significant interactions, but the stretched leg showed a trivial magnitude, main effect for time (F_(1,12)_ = 10.32, p = 0.007, 𝜂𝑝^2^ = 0.462) with a 6.1% (d = 0.15) greater PPT post-test, whereas the non-stretched, contralateral leg did not exhibit any significant differences. However, there were non-significant sex differences with the non-stretched leg male PPT (F_(1,12)_ = 4.22, p = 0.06, 𝜂𝑝^2^ = 0.260) exceeding females by 35.8% (d = 0.96). Furthermore, males also exceeded female values with shoulder PPT (F_(1,12)_ = 10.79, p = 0.007, 𝜂𝑝^2^ = 0.474) by 97.3% (d = 1.14).

### MVIC force

Each condition (including control) experienced significant force decreases from pre- to post-test (F_(3,36)_ = 10.79, p = 0.007, 𝜂𝑝^2^ = 0.181) (Table 1). The stretched leg exhibited a non-significant main effect for time (F_(1,12)_ = 2.64, p = 0.06, 𝜂𝑝^2^ = 0.619) with 7.3% (d = 0.31) MVIC force decreases from pre- to post test. A significant sex difference revealed that men exerted very large magnitude 38.2% (d = 1.71) significantly (F_(1,12)_ = 12.24, p = 0.004, 𝜂𝑝^2^ = 0.505) greater MVIC forces than women with their right (stretched) leg.

The non-stretched leg did not reveal any significant interactions. There was a main effect for time (F_(1,12)_ = 8.16, p = 0.014, 𝜂𝑝^2^ = 0.405) with 5.0% (d = 0.22) force decreases from pre- to post-test. In parallel with the stretched leg, the non-stretched leg also exhibited very large magnitude 36.5% (d = 1.87) greater male MVIC forces (F_(1,12)_ = 15.6, p = 0.002, 𝜂𝑝^2^ = 0.565). As expected, male shoulder MVIC forces significantly (F_(1,12)_ = 5.92, p = 0.03, 𝜂𝑝^2^ = 0.330) exceeded females by 47.6% (d = 1.51).

### MVIC EMG

There were no significant interactions for MVIC EMG. Comparable to the stretched leg MVIC forces main effect for time decreases, stretched leg MVIC EMG also exhibited a 6.1% (d = 0.14) trivial magnitude, non-significant pre- to post-test decrement (F_(1,12)_ = 4.09, p = 0.06, 𝜂𝑝^2^ = 0.255). Men displayed a large magnitude, 45.2% (d = 0.95) significantly (F_(1,12)_ = 4.97, p = 0.04, 𝜂𝑝^2^ = 0.293) higher stretched leg EMG values. However, there were no significant EMG differences with the non-stretched leg or shoulder.

### 30-s MVIC fatigue index

There were no significant interactions for either leg. The non-stretched leg demonstrated a main effect for condition (F_(3,36)_ = 4.36, p = 0.01, 𝜂𝑝^2^ = 0.267) with SS exhibiting significantly (p = 0.024, 59.8%, d = 0.81) greater MVIC force deficits effects than PNF (Table 2). The non-stretched leg also showed a non-significant EMG fatigue index main effect for condition (F_(3,36)_ = 2.28, p = 0.09, 𝜂𝑝^2^ = 0.160) with SS inducing 47.9% (d = 0.72) and 15.5% (d = 0.25) higher fatigue indexes than PNF and Dynamic stretching conditions respectively. Shoulder fatigue index for MVIC force or EMG did not achieve significance. The stretched (F_(1,12)_ = 12.57, p = 0.004, 𝜂𝑝^2^ = 0.512) and non-stretched legs (F_(1,12)_ = 4.94, p = 0.04, 𝜂𝑝^2^ = 0.292) demonstrated significant sex differences, with men experiencing 67.9% (d = 1.03) and 35.4% (d = 0.71) greater MVIC force fatigue indexes than women.

## Discussion

The primary findings of the present study were that following 30-seconds of SS, SS (post-test) elicited the greatest improvements in ROM in the stretched limb (8.1%) compared to control post-test (moderate magnitude). Significant SS-induced gains in ROM were observed with the stretched leg and non-significant increases with contralateral leg (non-significant: p = 0.08, large magnitude effect size), suggesting significant non-stretched lower limb ROM crossover effects might be observed with a larger sample size with more statistical power. There were no significant non-local effects on the right shoulder for any of the interventions on ROM, PPT, MVIC, or endurance. Despite increases in ROM, all conditions including the control resulted in decreased MVIC force from pre- to post-test with the stretched leg. PPT demonstrated a main effect for time (all interventions combined including control) increase with the stretched limb only. Clear sex differences were observed, with women demonstrating greater ROM and men exhibiting greater force, EMG activity, and fatigue indices.

The significant interaction showing that SS elicited greater ROM increases than control agreed with our initial hypothesis. The differences in ROM improvements between the interventions were small, which is consistent with the finding that all three modalities have been shown to increase ROM; however, there remains debate over which modality yields greater results in the acute timeframe [1–3,6]. Based on the prior literature, this ROM increase might initially be attributed to increases in stretch tolerance. However, there were no significant interactions showing greater PPT (pain onset). Alternatively, with all three stretch conditions, the stretch-induced tension placed on the tissues may have elicited thixotropic effects (decreased viscoelasticity) with reductions in muscle-tendon unit stiffness [1,2,8,28]. Another potential mechanism would be mediation through neural mechanisms, such as decreased motoneuron excitability and altered afferent input [1,2,8,24,29,30]. The 30 s of passive SS would have induced a disfacilitation of muscle spindle reflex activity (reduced Ia afferent discharge to the motoneurons) as the gamma efferent activation would have attempted to return the spindles to their optimal length. Thus, the reduced reflex activity would result in a more relaxed muscle. PNF and DS with their active contractions may have increased spindle reflex activity [1,2,8,29].

Another reason PNF and DS did not show meaningful changes compared to control may be that SS was the only condition to sustain the stretch for 30-seconds at the point of maximal discomfort. PNF reached 20 total seconds at the point of maximal discomfort (2 x 10-s of stretching, with 2 x 5-s durations contracting against the researcher). The 5-s isometric contraction was performed at the end ROM achieved after 10-s of static stretch. Hence, the muscle was still in an extended position when contracting, although the contraction may have removed it from a position of maximal discomfort and ROM. DS would not have met the same level of stretch intensity or duration at maximal ROM as either PNF or SS. DS would only have reached full ROM for minimal periods with each repetition (end point of each flexion and extension). The importance of remaining at the point of maximal discomfort throughout the stretch may be a mitigating factor in the superiority of SS over controls in this study. While the isometric contraction component would have shortened the muscle and placed additional stretch on the tendon, it did not exceed SS for improving ROM.

Although, the interaction was statistically non-significant (p = 0.08), there were numerous instances of individuals experiencing non-local increases in ROM. For example, with the SS, PNF, and DS conditions, there were 7, 5, and 5 participants (of the 14 participants) who experienced increased contralateral hip flexion ROM increases respectively. Only 1 participant showed increased pre- to post contralateral ROM increases with the control condition.

Since the contralateral leg was not stretched, the findings from these individuals are unlikely to be explained by mechanical responses. As mentioned, SS had the longest duration at the point of maximal discomfort. While longer stretch durations have been speculated to augment stretch tolerance [1,2,22,29], there was no significant PPT increases in the non-stretched leg. Furthermore, Hadjizazdeh Anvar et al. [21] reported that the spinal motoneurons and corticospinal excitability may not play a substantial role in non-local muscle’s ROM or force output responses. The lack of statistical significance (p = 0.08) but large magnitude effect size gains make a mechanistic interpretation difficult.

The present study’s main effect for time suggests that PPT increased regardless of the intervention, including the control condition. Similar findings were discovered for MVIC force and EMG with all conditions experiencing deficits. This may suggest that the PPT increases and MVC and EMG impairments are not solely attributable to the stretching protocols but instead also reflect a testing effect or carry-over effects from the collection of ROM and MVIC measurements. ROM testing required bringing the limbs to the point of maximal discomfort, and MVIC measurements required maximal-effort contractions, both of which may activate inhibitory pathways. Some studies have also suggested that increased blood circulation and thixotropic effects can induce pain-inhibiting effects in the muscle [10,31]. Further, PPT is a sensory or perceptual measure in which the participant indicates the onset of pain, and with repeated exposure, there may be a familiarization effect.

This finding is generally consistent with previous literature indicating that testing as well as prolonged acute stretching can impair force production and neuromuscular activation, particularly SS and PNF [1,8,23,29,32–34]. Proposed mechanisms underpinning MVIC testing-induced decreases in force include contractile fatigue (damage caused from pre-intervention ROM testing and/or MVICs, thus reducing contractile force capacity), and reduced central/efferent drive (i.e., reduction of muscle spindle feedback to motoneuron pool) [1,8,23,29,32–34]. The decrements observed in the present study might be explained by central fatigue arising from the pre-intervention MVICs. Intense cognitive activity can impair cognitive functioning or induce mental fatigue, which may negatively impact future performance [35,36]; thus the cognitive demands of sustained focus or concentration during ROM, PPT, MVIC measures may have contributed to the decreases in force observed in the stretched limbs. There was no significant decrease in MVIC force in the non-stretched limb.

Significant sex differences were observed in this study, with women demonstrating greater ROM in both the stretched and non-stretched leg for all stretching modes, and men exhibited higher MVIC force, EMG activity, and fatigue indices. These findings are consistent with the literature indicating females typically display greater flexibility, which may be due to differences in muscle mass and lower musculotendinous stiffness [1,29]. The greater force and EMG values observed in men are likely explained by similar reasoning: their larger muscle mass enables them to produce higher forces. The magnitude of these differences was substantial, which suggests sex-specific characteristics may play a large role in both flexibility and performance. The higher fatigue indices in men might reflect their greater absolute force production, which can likely result in greater relative declines across repeated maximal contractions.

Several limitations should be considered for this study. The limited sample size of 14 may limit the statistical power required to detect subtle interactions between conditions and sex, particularly those of the non-stretched limb effects. Although significance was achieved with some variables, none of the results were within the minimal detectable differences calculation and thus should be interpreted with caution. With this limited between sex participant population, a sex-stratified analysis is weakened and thus the study was not designed primarily for the detection of sex differences. However, it is a common and justified complaint that sport science lacks adequate representation of the female population. Although higher participant numbers would be preferable, we still feel that including women was important. Furthermore, participants were limited to a healthy, physically active university population, which may restrict generalizability. The 30-s intervention duration, which was meant to be uniform across the different interventions, was also a limitation. SS reached the full 30-s of maximum tolerable muscle lengthening, whereas the 30-s interventions of the other two modalities did not, which might have affected their outcomes. The lack of non-local effects observed in this study may also be attributable to the very acute stretching duration. Regarding the lack of testing order randomization, the 2–3 MVICs in the pre-test would undoubtedly have had thixotropic effects on the ROM and thus it was decided to place the MVIC last in order to minimize testing effects on ROM. The 2-minute rest period between the end of pre-testing and start of post-testing may have still retained residual MVIC fatigue effects and thus future research should lengthen the pre- to post-testing duration to minimize possible fatigue effects. Furthermore, as there was only one assessor in the lab, he was not blinded to the condition assignment. Future studies should employ blinding of assessors to condition.

## Conclusions

This study sought to investigate the acute local and non-local effects of various stretching modalities on ROM, PPT, and neuromuscular performance. The findings suggest that while passive ROM of the stretched leg may improve across all conditions, these gains can be accompanied by declines in MVIC force. The presence of these performance deficits within the control group suggests a potential testing effect, indicating that the pre-intervention data collection may have induced some central fatigue and habituation that overshadowed the isolated effects of the stretching interventions. Overall, 30-s was enough to elicit notable increases in ROM; however, it failed to produce any significant effects on the shoulder. This suggests that future research should investigate longer durations and higher intensities to identify which modality produces the most profound effects on ROM and PPT, both locally and non-locally. Additionally, providing longer recovery periods between pre- and post-test measurements may better isolate the acute effects of fatigue, and having more participants may increase statistical power, enabling a greater understanding of the effects, so larger sample sizes should be used.
